# YIPF2 regulates genome integrity

**DOI:** 10.1186/s13578-024-01300-x

**Published:** 2024-09-05

**Authors:** Xiao Zhang, Tao Wang

**Affiliations:** 1 Guangdong Provincial Key Laboratory of Stem Cell and Regenerative Medicine, Guangdong-Hong Kong Joint, Laboratory for Stem Cell and Regenerative Medicine, Guangzhou Institutes of Biomedicine and Health, Chinese Academy of Sciences, Guangzhou, China; 2 GIBH-HKU Guangdong-Hong Kong Stem Cell and Regenerative Medicine Research Centre, Guangzhou, China; 3 GIBH-CUHK Joint Research Laboratory On Stem Cell and Regenerative Medicine, Guangzhou, China; 4 University of Chinese Academy of Sciences, Beijing, China

## Abstract

Understanding of the mechanisms for genome integrity maintenance can help in developing effective intervention strategies to combat aging. A whole-genome RNAi screen was conducted to identify novel factors involved in maintaining genome stability. The potential target genes identified in the screening are related to the cell cycle, proteasome, and spliceosomes. Unexpectedly, the Golgi protein YIPF2 has been found to play a critical role in maintaining genome stability. The depletion of YIPF2 hinders the process of homologous recombination (HR) repair, which then triggers DNA damage response mechanisms, ultimately leading to cellular senescence. The overexpression of YIPF2 facilitated cellular recovery from DNA damage induced by chemotherapy agents or replicative senescence-associated DNA damage. Our findings indicate that only the intact Golgi apparatus containing YIPF2 provides a protective effect on genome integrity.

**Supplementary Information:**

The online version contains supplementary material available at 10.1186/s13578-024-01300-x.

## Introduction

The nuclear genome is constantly exposed to a variety of endogenous and exogenous factors, leading to the initiation and accumulation of diverse DNA damage and lesions within cells [1, 2]. DNA damage has been identified as the primary factor that contributes to the aging process [3–5]. Early studies indicated a relationship between excision-repair capability and longevity in a healthy population [6]. The repair capacity for double-strand breaks (DSB) is positively correlated with the maximum healthy lifespan in long-lived species [7].

Deficiencies in DNA repair enzymes, such as ERCC1 or XPG, in mice have been shown to lead to significant premature aging phenotypes [8–10]. Mutated DNA repair proteins have been associated with manifestations of accelerated aging. A cluster of proteins, such as BLM, WRN, and RECQL4, participated in the repair of DNA damage and the maintenance of genome stability. Mutations in these proteins was linked to human progeroid diseases, namely Bloom syndrome (BS), Werner syndrome (WS), and Rothmund-Thomson syndrome (RTS), respectively [11].

The molecular mechanisms responsible for DNA damage-induced senescence involved in the DNA damage response (DDR) activating ATR, ATM, and p53. These mechanisms inhibit cell growth by activating cyclin-dependent kinase inhibitors such as p16, p21, and p27, and by inducing hyperphosphorylation of the retinoblastoma protein [12].

Prior studies have demonstrated that genome instability can induce an inflammatory response through the cGAS-STING pathway [13, 14]. Senescent cells typically exhibit impaired genome integrity, characterized by the presence of micronuclei or cytoplasmic chromatin fragments (CCF) [15, 16]. The chronic inflammatory response is primarily thought to be caused by the activation of the cGAS-STING pathway, which is primarily mediated by the presence of cytoplasmic chromatin fragments (CCF) [15–19]. CCF is defined by a heterochromatin structure that contains H3K9me3 histone markers [15, 16]. Moreover, CCF also exhibits positivity for γH2A.X [15, 16], suggesting the involvement of double-strand breaks in the genesis of CCF [20]. Based on these observations, it is hypothesized that the presence of chromosomal DNA fragmentation (CCF) could serve as an indicator of the integrity of the nuclear genome.

Organisms have evolved multiple mechanisms to protect genome integrity by recognizing and repairing different forms of DNA damages [21]. The maintenance of genome integrity primarily relies on three crucial pathways: DNA damage repair systems, DNA replication, and chromosome separation during the process of mitosis [22, 23]. As mentioned above, dysregulation of these biological pathways can lead to a range of severe diseases, such as cancer, degenerative diseases, premature aging, and other abnormalities [24, 25]. Convergent researches have shown that genome integrity is compromised as individuals age, resulting in the accumulation of DNA lesions [26–28].In aged tissues, DNA damage can be triggered by inflammation associated with senescence-associated secretory phenotype (SASP) [29]. Numerous studies have revealed a decrease in both the expression levels and activity of DNA damage repair enzymes with aging. Considering these observations, it is reasonable to infer that enhancing DNA repair capacity may improve genome stability and delay the aging process. Several rejuvenation strategies, such as NAD + supplementation and caloric restriction, have been recognized for their capacity to enhance DNA repair [30, 31]. The overexpression of Sirt6 also restored DNA repair capacity and improved genome stability [32]. However, the upregulation of particular DNA double-strand break (DSB) repair enzymes often leads to genomic instability or hinders the efficiency of homologous recombination (HR) [33, 34]. DNA damage repair pathways consist of a variety of enzymes or factors whose activities are tightly controlled in response to different forms of damage. Hence, delaying the aging process by enhancing DNA repair solely through the upregulation of the activity of one or multiple genes poses a considerable challenge [5]. Based on these observations, it is worthwhile to explore new regulators of DNA repair systems to enhance our understanding of genome stability maintenance. In this study, we opted to utilize CCF counts as the readout to discover novel regulators for genome integrity by employing a whole-genome RNAi library. Through the screening process, the Golgi-resident protein YIPF2 was discovered to play a crucial role in maintaining genome integrity.

## Results

### Identification of novel factors regulating genome integrity by RNAi screening

**Fig. 1:**
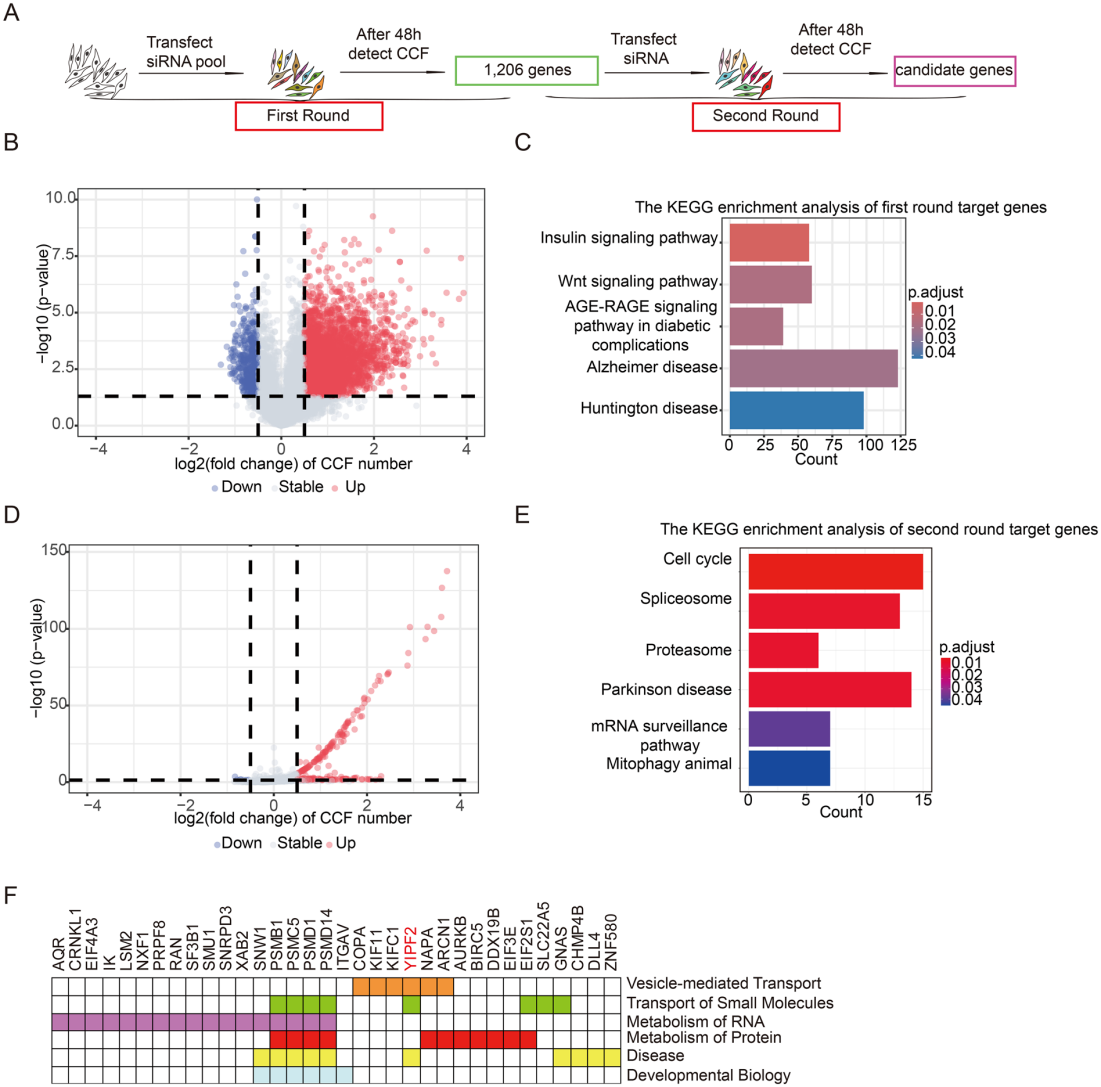
Genome-wide siRNAs screening identifies YIPF2 as a novel protein involved in genome integrity. **A** Workflow of the genome-wide siRNAs screening. IMR90 cells were transfected with siNC or a mixed four siRNAs. 1206 genes were screened out in the first-round screening. The second-round screening for these 1206 genes was carried out using the same procedure. **B** Volcano plot of CCF fold changes (Log2FC) in the first-round screening. **C** KEGG analysis of 1206 candidate genes identified in the first-round screening. **D** Volcano plot of CCF Log2FC in the second-round screening. **E** KEGG analysis of candidate genes in the second screening. **F** List of genes in the siRNA screening that are associated with genome integrity

A genome-wide siRNA screening was conducted in IMR90 cells using CCF as a DNA damage indicator to identify potential regulators maintaining genome integrity. After a 72 h delivery of the siRNA library, DNA damage was assessed by quantifying CCF number, which are γH2A.X and H3K9me3 double positive [17]. A total of 1206 genes were identified to be involved in the regulation of genome integrity, as indicated by an increase in CCF counts following siRNA transduction (Fig. 1A, B). The genes were enriched in pathways related to insulin signaling pathway, Wnt signaling pathway, AGE-RAGE signaling pathway in diabetic complications, Alzheimer disease, Huntington disease, as determined by Kyoto Encyclopedia of Genes and Genomes (KEGG) (Fig. 1C). To validate the candidate hits from the initial round of screening, a second round was conducted using four individual siRNAs targeting these 1206 genes, following the same procedure and protocol (Fig. 1A). The data indicated that approximately 100 genes were validated and they were enriched in pathways related to DNA damage, such as the cell cycle, spliceosome, proteasome, Parkinson’s disease, mRNA surveillance pathway, and mitophagy (Fig. 1D, E). Remarkably, our screening identified new targets like LSM2, ARCN1, and YIPF2, not previously linked to DNA damage in the literature (Fig. 1F). YIPF2 belongs to the Yip domain family (YIPF), which comprises seven Golgi-resident proteins, YIPF1 to YIPF7. The protein is believed to have five transmembrane segments, with an N-terminal segment facing the cytoplasm and a short C-terminal segment facing the Golgi lumen to facilitate protein transport [35]. However, only YIPF2 depletion caused CCF foci increase (Fig. S1A). Therefore, we selected YIPF2 for further investigation to determine how Golgi-localized YIPF2 regulates genome integrity.

### YIPF2 deficiency impairs genome integrity

**Fig. 2:**
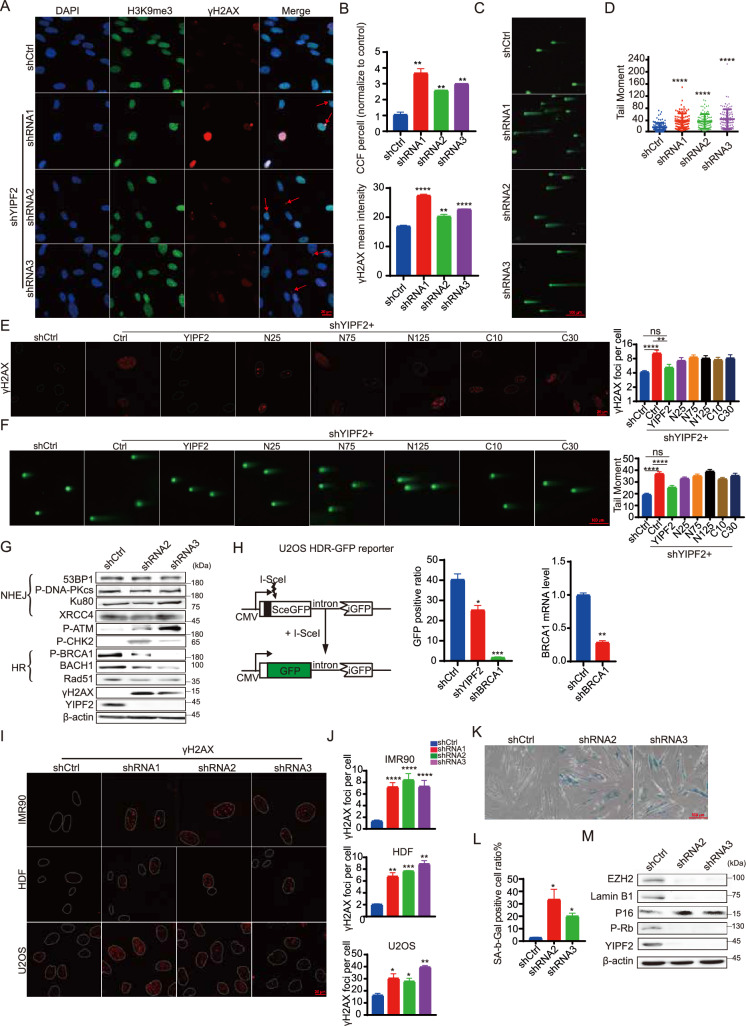
YIPF2 depletion impaired genomic integrity. **A** Detection of CCF foci in control and YIPF2-depleted IMR90 cells. The red arrow marks CCF. Scale bars, 20 μm. **B** Quantification of (**A**): the number of CCF foci per cell and nuclear γH2A.X signaling intensity (n ≥ 100). Error bars indicate mean ± SEM of three independent experiments. P values were calculated using a one-tailed Student’s t-test (**p < 0.01, ****p < 0.0001). **C** Detection of neutral comet assay in the control and YIPF2-depleted IMR90 cells. Scale bars, 100 μm. **D** Quantification of (**C**): the tail moment of each cell (n ≥ 100). Error bars indicate mean ± SEM of three independent experiments. P values were calculated using a one-tailed Student’s t-test (****p < 0.0001). **E** Detection of γH2A.X foci in the control and full-length or truncated YIPF2 construction in YIPF2-depleted IMR90 cells. Scale bars, 20 μm.** F**. Quantification of the tail moment of each cell (n ≥ 100). Error bars indicate mean ± SEM of three independent experiments. P values were calculated using a one-tailed Student’s t-test (****p < 0.0001). **G** Immunoblotting analysis of the DDR markers in control and YIPF2-depleted IMR90 cells. **H** Evaluation of the effect of YIPF2 on DNA damage repair using HDR-GFP reporter. Diagram of the U2OS HDR-GFP reporter (left). HDR activity in the control and YIPF2-depleted U2OS HDR-GFP cells were examined, knockdown of BRCA1 was chose as a positive control (middle). The BRCA1 mRNA level was confirmed by RT-PCR (right). Error bars indicate mean ± SEM of three independent experiments. P values were calculated using a one-tailed Student’s t-test (*p < 0.05, **p < 0.01). **I** Detection of γH2A.X foci in control and YIPF2-depleted IMR90 cells, HDF cells and U2OS cells. Scale bars, 20 μm. **J** Quantification of (**I**): the number of γH2A.X foci per cell (n ≥ 100). Error bars indicate mean ± SEM of three independent experiments. P values were calculated using a one-tailed Student’s t-test (****p < 0.0001, ***p < 0.001, **p < 0.01, *p < 0.05). **K** Detection of SA-β-gal activity in control and YIPF2-depleted IMR90 cells. Scale bars, 100 μm. **L** Quantification of (**K**): the percentage of SA-β-gal staining positive cells (n ≥ 100). Error bars indicate mean ± SEM of three independent experiments. P values were calculated using a one-tailed Student’s t-test (*p < 0.05). **M** Immunoblotting analysis of the cellular senescent markers in control and YIPF2-depleted IMR90 cells

To validate the screening results, we utilized short hairpin RNAs (shRNAs) to interfere with YIPF2 expression (Fig. S2A). The levels of H3K9me3 and γH2A.X-positive CCF and nuclear γH2A.X intensity were significantly elevated in YIPF2-depleted cells (Fig. 2A and B). Genome integrity in these cells was compromised, as indicated by the results of the neutral comet assay (Fig. 2C and D). To confirm the phenotypes of YIPF2-depleted cells, we complemented shRNA-knockdown cells with full-length and different truncated YIPF2 (Fig. S2B). The different constructions were confirmed by Western blotting (Fig. S2C). Only full-length YIPF2 could rescued the double-strand breaks (DSBs) and genome integrity phenotypes, as indicated by γH2A.X immunostaining and neutral comet assay (Fig. 2E and F), which indicated that both N-terminal and C terminal were important for YIPF2 functions. These results suggest that YIPF2 is a novel protein involved in maintaining genome integrity.

A significant increase in double-strand breaks (DSBs) was observed in the absence of YIPF2, therefore, we examined whether YIPF2 regulates DNA damage repair. In mammalian cells, there are two major pathways for double-strand break (DSB) repair – non-homologous end joining (NHEJ) and homologous recombination (HR). The choice between the two pathways depends on the phase of the cell cycle and DSB ends [36]. Previous studies have shown that BRCA1 and 53BP1 are involved in determining whether non-homologous end joining (NHEJ) repair or homologous recombination (HR) repair is utilized to repair DSB sites [37–39]. We detected the levels of core proteins in the repair complex after YIPF2 knockdown. It was observed that 53BP1, phosphorylated DNA-PKcs, Ku80, and XRCC4 remained unchanged in the YIPF2 knockdown cells. However, phosphorylated BRCA1, BACH1, and RAD51 were apparently decreased (Fig. 2G). Consistently, DNA double-strand break sensors, the phosphorylated ATM and CHK2, were upregulated in these cells (Fig. 2G). Subsequently, we used a well-established homology-directed repair GFP (HDR-GFP) reporter [40], to measure HR repair activity. The YIPF2 knockdown reduced the number of GFP-positive cells by 40% compared to the control group, while knockdown of BRCA1 almost completely blocked HR repair (Fig. 2H). These data suggested that HR repair was inhibited in the absence of YIPF2.

Furthermore, we checked the effects of YIPF2 on DSB repair in zeocin treated cells, which mimic radiation-induced DSB damage [41]. It was found that YIPF2 did not affect the co-localization of 53BP1 with γH2A.X (Fig. S2D, S2E); however, the recruitment of BRCA1 to chromatin was apparently reduced in YIPF2-depleted cells (Fig. S2F, S2G).

We also knocked down the protein in two other cell lines, including human dermal fibroblast (HDF) and U2OS cells. The data showed that YIPF2 depletion resulted in a significant increase in γH2A.X foci in the two cell lines (Fig. 2I and J), suggesting that the effect is independent of cell type. Furthermore, we found that only depletion of YIPF2 among the YIPF family proteins resulted in a significant accumulation of nuclear γH2A.X foci (Fig. S2H, S2I). These results demonstrated that YIPF2 was involved in maintaining genome integrity by regulating HR repair.

Because DNA damage has been shown to induce senescence [42–44], we performed several assays to evaluate the senescence status of YIPF2-depleted cells. Around 8 days after shRNAs transduction, cells ceased proliferation, as indicated by EdU staining (Fig. S2J and S2K). These YIPF2-depleted cells gradually entered senescence, as evidenced by flat and enlarged nuclei, and positive staining for senescence-associated β-galactosidase (SA-β-gal) activity (Fig. 2K and L). Moreover, senescence-associated markers, including EZH2 [45, 46], Lamin B1 [47, 48] and phosphorylated Rb, decreased, while p16 [49] increased in YIPF2-depleted cells (Fig. 2M). These results demonstrated that YIPF2 deficiency drove cells toward senescence mediated by DNA damage.

### YIPF2 overexpression promotes DNA damage repair

**Fig. 3:**
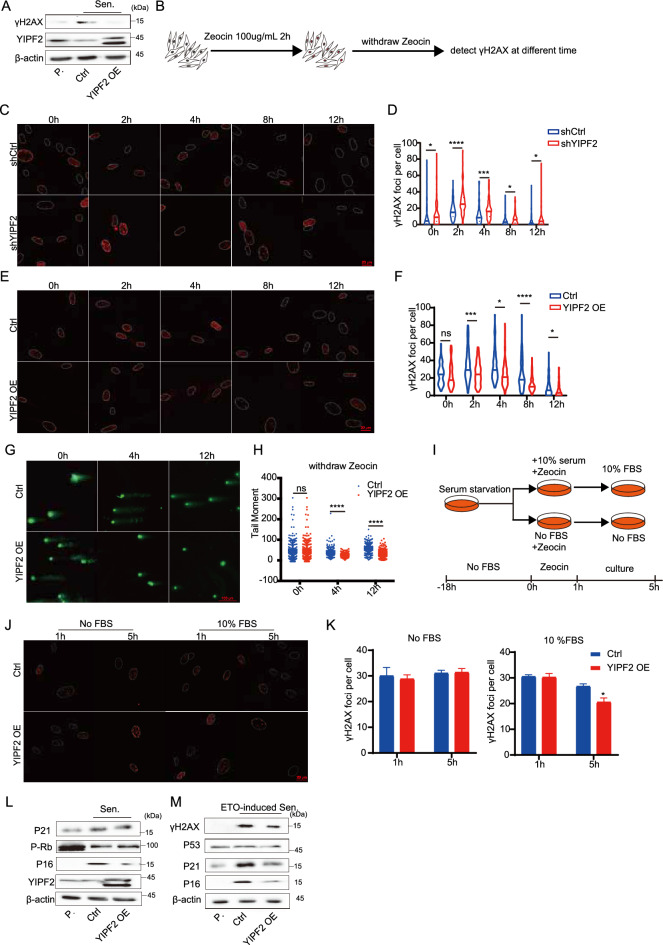
Overexpression of YIPF2 promotes genomic integrity and DDR. **A** Immunoblotting analysis of the γH2A.X level and YIPF2 level in proliferating or senescent cells and YIPF2 overexpression in senescent cells (P. represents proliferating cells; Sen. represents senescent cells). **B** Schematic of Zeocin treatment experimental workflow was shown. Cells were treated with 100 μg/mL Zeocin for 2 h and continued cultivation for different times and γH2A.X foci were examined. **C** Detection of γH2A.X foci after 0, 2, 4, 8, 12 h withdrawal of Zeocin in control and YIPF2-depleted IMR90 cells. Scale bars, 20 μm. **D** Quantification of (**C**): the number of γH2A.X foci per cell (n ≥ 100). Error bars indicate mean ± SEM of three independent experiments. P values were calculated using a one-tailed Student's t-test (****p < 0.0001, ***p < 0.001, *p < 0.05). **E** Detection of γH2A.X foci after 0, 2, 4, 8, 12 h withdrawal of Zeocin in control and YIPF2 overexpressing IMR90 cells. Scale bars, 20 μm. **F** Quantification of (**E**): the number of γH2A.X foci per cell (n ≥ 100). Error bars indicate mean ± SEM of three independent experiments. P values were calculated using a one-tailed Student's t-test (****p < 0.0001, ***p < 0.001, *p < 0.05). **G** Detection of neutral comet assay after 0, 4, 12 h withdrawal of Zeocin in control and YIPF2 overexpressing IMR90 cells. Scale bars, 100 μm. **H** Quantification of (**G**): the tail moment of each cell (n ≥ 100). Error bars indicate mean ± SEM of three independent experiments. P values were calculated using a one-tailed Student’s t-test (****p < 0.0001). **I** Schematic of serum starvation experimental workflow. Cells were cultured without serum for 18 h. Then the cells were treated with 100 μg/mL Zeocin for 1 h under conditions of 10% FBS or no FBS and continued cultivation with 10% FBS or no FBS for another 4 h. **J** Detection of γH2A.X foci under conditions of no FBS or 10% FBS in control and YIPF2 overexpressing IMR90 cells. Scale bars, 20 μm. **K** Quantification of γH2A.X foci per cell in Fig. 3 J (n ≥ 100). Error bars indicate mean ± SEM of three independent experiments. P values were calculated using a one-tailed Student’s t-test (*p < 0.05). **L** Senescent markers in control and YIPF2 overexpression IMR90 cells were detected using western blotting. **M** Immunoblotting analysis of the DNA damage and cellular senescence markers in control and YIPF2 overexpressing IMR90 cells with ETO treatment

In light of the DNA damage accumulation resulting from YIPF2 deficiency, we aimed to investigate whether YIPF2 overexpression can enhance genome integrity using a replication senescence model and a zeocin-induced DNA damage model. Indeed, we found that the level of γH2A.X in senescent IMR90 cells significantly increased because DNA damage repair capacity decreased along with senescence, as shown in previous studies [50] (Fig. 3A). Overexpression of YIPF2 could reduce senescence-associated γH2A.X formation (Fig. 3A). Zeocin treatment induced DNA damage can be repaired by the endogenous DNA damage repair system as indicated by γH2A.X staining and comet assay (Fig. 3B–H). Our study showed that YIPF2 depletion disrupted the repair capacity (Fig. 3C and D); but overexpression of YIPF2 promoted DNA damage repair (Fig. 3E–H).

HR repair is intimately related with S phase; to determine whether the cell cycle also influences YIPF2-mediated DNA repair, we evaluate repair efficiency in G1 and S phases. Cell cycle was synchronized by removing serum from the culture media for 18 h to induce cell in the G1 phase, subsequently,10% serum was used to promote cell to S phase [51], simultaneously, zeocin was employed to induce DNA damage (Fig. 3I). Around 5 h, EdU staining revealed that around 10% of the cells were in the S phase (Fig. S3A). YIPF2 was observed to enhance DNA damage repair only in the S phase, not in the G1 phase (Fig. 3J and K). The data suggests that YIPF2 is critical for genomic integrity.

Based on the observed effect of YIPF2 overexpression on DNA damage repair, we evaluated the impact of YIPF2 overexpression on cell senescence. YIPF2 overexpression significantly reduced the number of SA-β-gal-positive cells in both the replication senescence model and the zeocin-induced senescence model. Similarly, the protein levels of cellular senescence markers and DNA damage response were reduced in YIPF2-overexpressing cells in these two models (Fig. 3L and M, S3B and S3C). These results demonstrate that the overexpression of YIPF2 promotes DNA damage repair and delays cell senescence.

### YIPF2 regulates DNA replication relevant genes

**Fig. 4:**
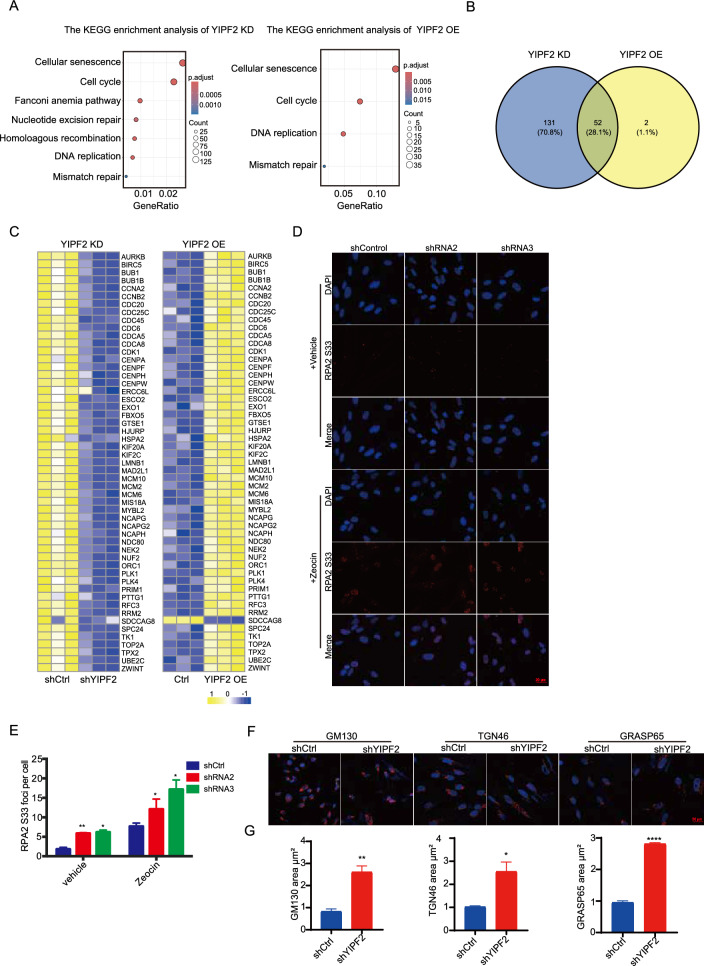
YIPF2 regulated DNA replication genes. **A** KEGG analysis of differentially expressed genes (DEG) for YIPF2-depleted cells (left panel) and YIPF2 overexpressing cells (right panel). **B** Venn diagram analysis of DEGs including cell cycle and DNA replication relevant genes in YIPF2-depletion cells and YIPF2 overexpressing cells. **C** Heatmap analysis of the co-regulated DEGs in YIPF2-depletion cells (left panel) and YIPF2 overexpressing cells (right panel). **D** Detection of pRPA2 S33 foci in control and YIPF2-depleted IMR90 cells treated with DMSO or Zeocin. Scale bars, 20 μm. **E** The number of pRPA2 S33 foci per cell was examined (n ≥ 100). Error bars indicate mean ± SEM of three independent experiments. P values were calculated using a one-tailed Student's t-test (**p < 0.01, *p < 0.05). **F** Detection of Golgi apparatus morphology in control and shYIPF2 cells. Immunostaining of GM130, TGN46 and GRASP65. **G** The area of Golgi was quantified by ZEN (n ≥ 100). The relative ratio is shown. Error bars indicate mean ± SEM of three independent experiments. P values were calculated using a one-tailed Student’s t-test (****p < 0.0001, **p < 0.01, *p < 0.05)

To understand how YIPF2 regulates DNA damage repair, we investigated differentially regulated expression genes (DEGs) in response to YIPF2 depletion or overexpression using RNA-Seq data. KEGG analysis of DEG genes revealed that several pathways, including cell cycle, cellular senescence, DNA replication, and mismatch repair, were commonly enriched in the two RNA-Seq datasets (Fig. 4A, left and right panels). The transcriptome analysis results were consistent with DNA damage phenotypes in response to YIPF2 expression.

We observed that BRCA1, long-range end-resection factor exonuclease 1 (EXO1), the DNA crosslinking helicase FA complementation group D2 (FANCD2), and DNA ligase 1 (LIG1), which are involved in HR repair, decreased in YIPF2 depletion cells (Fig. S4A). This consistents with the findings of impaired DNA damage repair capacity due to YIPF2 knockdown (Fig. 3C).

Moreover, it was found that cell cycle and DNA replication-related genes were closely regulated by the level of YIPF2, as demonstrated by gene interference and gene overexpression (Fig. 4A). We overlapped these gene sets and found that a variety of proteins (52 proteins) were common targets, including MCM family proteins, CDC family proteins, and centromere proteins (Fig. 4B and C). Specifically, MCM proteins, which are involved in replication by forming replicative DNA helicase motor [52], were commonly regulated by YIPF2 deficiency or overexpression (Fig. 4C). The analysis implies that YIPF2 may be essential for DNA replication. Indeed, we found that depletion of YIPF2 caused a significant increase in pRPA2 S33 foci, which is an indicator of DNA replication stress [53, 54] (Fig. 4D, E and S4B). Additionally, the overexpression of YIPF2 reduced the level of pRPA2 S33 induced by zeocin or replicative senescence (Fig.S4B). It was believed that R-loops are usually formed in the presence of DNA replication stress [55, 56]. Fanconi Anemia proteins, which protect genome integrity by removing R-loops [57], were also enriched in the list (Fig.S4A). Therefore, we examined whether the R-loop level changed following alterations in YIPF2 expression levels. The S9.6 level significantly increased in YIPF2-depleted cells but decreased in YIPF2-overexpressing cells, as detected by dot blotting (Fig. S4C). As expected, the S9.6 signal was eliminated when samples were exposed to RNase H, a nuclease specific for R-loop [58] (Fig. S4C). These data suggest that YIPF2 regulates genome integrity through HR repair and DNA replication.

YIPF2 is known to localize in the Golgi apparatus. Our data also showed that YIPF2 doesn’t appear in the nucleus (Fig. S4D and S4E). Therefore, we aimed to investigate whether YIPF2-depletion induced DNA damage associate with alterations in the Golgi structure. The results showed that knocking down YIPF2 led to an increase in cells with dispersed Golgi, as confirmed by TGN46, GM130, and GRASP65 staining (Fig. 4F and G). As previously reported [59], DNA damage triggers Golgi dispersal. YIPF1-depletion also led to an increase in cells with dispersed Golgi, however, there was no DNA damage occurred as indicated by γH2A.X staining (Fig.S4F and S4G). The data suggested that Yip family were crucial for maintaining intact Golgi structure, but it is unclear if YIPF2 performs any unique tasks including genome integrity maintenance in the Golgi structure that are not shared by other YIPF proteins. On the other hand, Golgi resided YIPF2 is necessary for full function of YIPF2 mediated DNA damage repair (Fig. 2E, F).

Taken together, our results demonstrate that YIPF2 is crucial for genome stability through DNA replication and HR repair by regulating the transcription of relevant genes including DNA damage repair and DNA replication genes (Fig. S4H).

## Discussion

Maintenance of genome integrity is crucial for preventing premature senescence. Understanding the mechanisms of DNA damage repair could contribute to interventions for aging and age-associated diseases.

In this study, we used CCF as a readout to screen factors regulating genome stability. Unexpectedly, the Golgi-localized protein YIPF2 was identified as a novel regulator that maintains genome integrity.

YIPF2 belongs to the YIP family, which consists of seven proteins. YIPF family proteins have five transmembrane domains. The N-terminal regions face the cytoplasm, and a short C-terminal region resides in the Golgi lumen [60]. The family of proteins plays vital roles in intracellular vesicular transport [61–64]. Previous studies have shown that YIPF6 forms complexes with YIPF1 and YIPF2 to regulate glycan synthesis [35]. However, in the study, we found that only YIPF2 depletion caused DNA damage and genome instability (Fig. S2H), suggesting that the regulatory role of YIPF2 in DNA damage is specific to the protein.

Mammalian cells use non-homologous end joining (NHEJ) and homologous recombination (HR) to repair DSB [36, 65, 66], Depletion of YIPF2 resulted in a decrease in BRCA1 protein levels, impairing HR repair (Fig. 2G and S2F). Conversely, overexpression of YIPF2 promoted DNA damage repair and genomic integrity (Fig. 3A–H). Previous research has shown that DNA damage triggers Golgi dispersal to regulate cell survival [59]. Thus, it is possible that the Golgi apparatus regulates nuclear genome stability. The influence of cytoplasmic Golgi on DNA damage repair and genome integrity remains unknown in details. However, the YIP family member YIPF1 depletion didn’t induce DNA damage (Fig. S4F and S4G). Understanding the regulatory role of YIPF2 in DNA damage repair may help elucidate the crosstalk between the cytoplasm and the nucleus in response to DNA damage.

## Materials and methods

### Cell culture and siRNA screening

The cells were cultured in DMEM media with 10%FBS and 1% penicillin/streptomycin supplementation. IMR90 cells and HDF cells were cultured in an incubator at 37 ℃, 5% CO_2_, 3% oxygen. And other cells were cultured at 37 ℃, 5% CO_2_. The siRNA screening was performed as previously described methods at Chemical Biology Core Facility in CEMCS, CAS [67]. The whole genome human ON-TARGETplus siRNA Library (Horizon) was used to perform the screening. The first siRNA screening was performed with four replicates in 384-well plates. Around 18,000 siRNA pools (four siRNAs targeted one gene) were transfected into IMR90 cells using Lipofectamine^™^ RNAiMAX (Thermo Fisher) according to the provided protocol. After 72 h of transfection, cells were fixed to perform immunostaining with indicated antibodies, and were analyzed by High Content Screening (GE IN Cell Analyser 6500HS). Cells with three or more cytoplasmic chromatin fragments which are H3K9me3 and γH2A.X double positive was defined as CCF positive cells. 1206 genes were screened out in the first screening. The second screening was performed using individual siRNA with same procedure. Scramble siRNA controls were setup in each plate during screening. The number of CCF for siRNA controls in each plate were used to normalize data.

### Plasmids and lentivirus production

For the expression of YIPF2 protein, YIPF2 ORF cDNA and truncated YIPF2 mutants were amplified from cDNA. The PCR product was ligated into HpaI and BamHI site of pLVX expression vector via ClonExpress II One Step Cloning Kit (Vazyme). ShCtrl and YIPF2-specific 21 nt shRNA sequences were cloned into AgeI and EcorI site of pLKO plasmid. PLKO-shCtrl (Target Sequence: CCTAAGGTTAAGTCGCCCTCG), PLKO-YIPF2-shRNA1 (Target Sequence: AGCTACTATCAGAGCTTCTTT), PLKO-YIPF2-shRNA2 (Target Sequence: CATGGGCTGTAAGTTGTACTT), PLKO-YIPF2-shRNA3 (Target Sequence: CTTCAGCTACTATCAGAGCTT), PLKO-shBRCA1 (Target Sequence: GAGTATGCAAACAGCTATAAT), PLKO-YIPF1-shRNA1 (Target Sequence: CGTACCATTATGTGCCCGAAT), PLKO-YIPF1-shRNA2 (Target Sequence: GTGACAATTGTGTTGCTCCAT). All shRNAs were synthetized in Genewiz. Lentivirus were prepared with second generation packing system. Briefly, psPAX2, pMD2.G, and transfer vectors were co-transfected into 293 T cells with polyethylenimine (PEI, linear MW 40 000, Yeasen). Lentivirus was collected 48 h after transfection.

### DNA damage induction

IMR90 cell lines were treated with Zeocin (100 μg/mL, Thermo Fisher) for 2 h; IMR90 cell lines were treated with ETO (0.5 μg/mL, MedChemExpress) for 5–7 days to induce DNA damage or cell senescence.

### Immunostaining

Cells cultured on coverslips were fixed with 4% paraformaldehyde at room temperature (RT) for 15 min followed by permeabilized with 0.3% Triton X-100 at RT for 30 min. Cells were blocked with 10% goat serum. Primary antibody was incubated at 4 ℃ overnight followed by incubation with fluorescence-labeled secondary antibodies (ThermoFisher) and DAPI for 1 h at RT and visualized by fluorescence microscopy. The following antibodies for immunostaining: γH2A.X (1:200, Santa Cruz, sc-517348), H3K9me3 (1:200, Abcam, ab8898), γH2A.X (1:500, CST, 9718), BRCA1 (1:200, Santa Cruz, sc-6954), 53BP1 (1:500, CST, 4937), p-RPA2 S33 (1:200, Bethyl, A300-246A).The images were acquired Zeiss Axion Observer 7 and Zeiss LSM 800 microscope. For quantification of the number of foci, over 100 cells were counted in every experiment and data from three independent experiments are shown.

### Neutral comet assay

The assay was performed as described method in detail [68]. Briefly, 1 × 10^5^ cells were added to molten low-melting agarose 37 ℃at a ratio of 1:10 and lysis at room temperature for 30 min. Then cells were incubated in pre-chilled neutral electrophoresis buffer at 4 ℃ for 1 h and 21 V electrophoresis for 30 min. Washed the slides with ddH_2_O twice and 70% ethanol for 5 min at room temperature and air-dry the slides. Add diluted SYBR^®^ Gold solution and incubated for 30 min. Image were taken with fluorescence microscope and analyzed with CaspLab software. For quantification of the tail moment, over 100 cells were counted in every experiment and data from three independent experiments are shown.

### Western blotting

Protein was extracted with RIPA Lysis Buffer System (Beyotime, P0013C) for SDS-PAGE electrophoresis. After blocking with 5% milk or BSA buffer for 1 h, the membranes were incubated with primary antibody at 1:1000 dilution overnight at 4 °C. After washing with PBS containing 0.1% Tween-20, membranes were incubated with secondary antibody targeting either anti-rabbit (Abcam, ab6721) or anti-mouse (Abcam, ab6708) at 1:3000 dilution for 1 h and signals were detected with BeyoECL western blotting substrate (Beyotime, P0018FM) and signal was detected by chemiluminescence. The following antibodies for western blot: p-ATM (CST, 5883), p-CHK2 (CST, 2197), p-BRCA1 (CST, 9009), p-DNA-PKcs (Abcam, ab18192), Rad51(Novus, NB100-148), Ku80 (Abclonal, A12338), XRCC4 (Abclonal, A1677), BACH1 (Abclonal, A5393), P53 (Proteintech, KHC0079), P21 (Proteintech, 10355-1-AP), p-RB1 (CST, 8516), Lamin B1 (Proteintech, 12987-1-AP), YIPF2 (ThermoFisher, PA5-54112), EZH2 (CST, 5246), β-actin (Abclonal, AC026), p-RPA2 S33 (Bethyl, A300-246A).

### RT-qPCR and data analysis

Total RNA was prepared using RNAiso Plus (TaKaRa, 9109) and cDNA was sunthesized from 1 μg total RNA using HiScript II Q Select RT SuperMix for qPCR (Vazyme, R233-01). YIPF1 qPCR-U: TCCAGATCTCTATGGCCCCTT, YIPF1 qPCR-L: GGAAACCCCAGAGTGCAAGA, BRCA1 qPCR-U: GAAACCGTGCCAAAAGACTTC, BRCA1 qPCR-L: CCAAGGTTAGAGAGTTGGACAC, β-actin qPCR-U: AGAAAATCTGGCACCACACC, β-actin qPCR-L: AGAGGCGTACAGGGATAGCA. Accumulation of PCR products was monitored in real time by measuring the level of fluorescence. Results were analysed by the ΔΔCt method and normalized to β-actin to determine relative fold changes in gene expression,

### SA-β-galactosidase staining

Cell senescence evaluation for IMR90 was performed with a Senescence β-Galactosidase Staining Kit (Beyotime, C0602) according to the provided protocol. Images were taken with microscopy. For quantification, over 100 cells were counted in every experiment and three independent experiments were shown.

### EdU assay

Cells grown on coverslips were treated with 10 μM EdU for 4 h and performed with an Edu assay kit (Beyotime, C0071L) according to the provided protocol. Images were taken with fluorescence microscopy and analyzed with Image J software. For quantification, over 100 cells were counted in every experiment and three independent experiments were shown.

### Quantification of Golgi structure

The Golgi structure stains were quantified the area occupy by ZEN software.

### S9.6 dot blot

Cells were lysed using 10% SDS and in 25 mM Tris–HCl (pH8.0) and 5 mM EDTA at 37 ℃ overnight with proteinase K. The DNA-RNA hybrids were extracted with phenol:chloroform:isoamyl alcohol (25:24:1, pH8.0). 200 ng samples were loaded to the nylon membranes with 1/10 volume Ammonium oxalate. Before blocking, the membranes were crosslinked using UV light (1200 μJ × 100) and stained with methyl blue. After blocking with 5% milk for 1 h, the membranes were incubated with S9.6 antibody (Millipore, MABE1095) at 1:1000 dilution overnight at 4 °C. After washing with PBS containing 0.1% Tween-20, membranes were incubated with secondary antibody anti-mouse (Abcam, ab6708)1:3000 dilution for 1 h and signals were detected with BeyoECL western blotting substrate (Beyotime, P0018FM) and signal was detected by chemiluminescence.

## Supplementary Information

Below is the link to the electronic supplementary material.

Supplementary file1 (XLSX 8812 KB)

## Data Availability

All data supporting the findings of this study are available within the paper and its Supplementary Table 1. Experimental materials are available upon request.
